# Chronic Glucocorticoid Exposure Induces Depression-Like Phenotype in Rhesus Macaque (Macaca Mulatta)

**DOI:** 10.3389/fnins.2019.00188

**Published:** 2019-03-08

**Authors:** Dongdong Qin, Zhifei Li, Zhaoxia Li, Limin Wang, Zhengfei Hu, Longbao Lü, Zhengbo Wang, Yun Liu, Yong Yin, Zhaofu Li, Xintian Hu

**Affiliations:** ^1^Key Laboratory of Animal Models and Human Disease Mechanisms of the Chinese Academy of Sciences and Yunnan Province, Kunming Institute of Zoology, Chinese Academy of Sciences, Kunming, China; ^2^Yunnan University of Chinese Medicine, Kunming, China; ^3^Department of Rehabilitation, Kunming Children’s Hospital, Kunming, China; ^4^Yunnan Key Laboratory of Primate Biomedicine Research, Institute of Primate Translational Medicine, Kunming University of Science and Technology, Kunming, China; ^5^Kunming Primate Research Center, Kunming Institute of Zoology, Chinese Academy of Sciences, Kunming, China; ^6^Department of Rehabilitation Medicine, the Fourth Affiliated Hospital of Kunming Medical University, Kunming, China; ^7^Center for Excellence in Brain Science and Intelligence Technology, Chinese Academy of Sciences, Shanghai, China

**Keywords:** chronic, glucocorticoid, HPA axis, depression, rhesus macaque

## Abstract

It has long been observed in humans that the occurrence of depressive symptoms is often accompanied by the dysfunction of hypothalamic-pituitary-adrenal (HPA) axis. The rodent experiments also showed that chronic corticosterone exposure could induce depression-like phenotype. However, rodents are phylogenetically distant from humans. In contrast, non-human primates bear stronger similarities with humans, suggesting research on primates would provide an important complement. For the first time, we investigated the effects of chronic glucocorticoid exposure on rhesus macaques. Seven male macaques were selected and randomized to glucocorticoid or vehicle groups, which were subjected to either prednisolone acetate or saline injections, respectively. The depression-like behaviors were assessed weekly, and the body weights, HPA axis reactivity, sucrose solution consumption and monoaminergic neurotransmitters were further compared between these two groups. The glucocorticoid group was not found to display more depression-like behaviors than the vehicle group until 7 weeks after treatment. Chronic glucocorticoid exposure significantly decreased the levels of cortisol determined from blood (a biomarker for acute HPA axis reactivity) but increased the hair cortisol concentrations (a reliable indicator of chronic HPA axis reactivity) compared with controls. The glucocorticoid group was also found to consume less sucrose solution than controls, a good manifestation of anhedonia. This could be possibly explained by lower dopamine (DA) levels in cerebrospinal fluid induced by chronic glucocorticoid treatment. The results presented here indicate that chronic glucocorticoid exposure could disturb both the acute and chronic HPA axis reactivity, which eventually disturbed the neurotransmitter system and led monkeys to display depression-like phenotype.

## Introduction

According to the World Health Organization (WHO), depression is estimated to become the second leading burden of illness in the world by 2020 (Mathers and Loncar, 2006). The core symptoms of depression include low mood and anhedonia (i.e., lack of interests in pleasurable activities), but it is always accompanied by a complex cluster of clinical symptoms that may include weight changes, sleep disturbances, psychomotor agitation or retardation, loss of energy, feelings of worthlessness, difficulty concentrating, and/or recurrent thoughts of death (American-Psychiatric-Association, 2013). In addition, depression can also cause increased physical illness, decreased social functioning, and a high mortality rate (Nemeroff, 1998). As is the case with other affective disorders, depression also has both a genetic and environmental basis. Twin studies revealed that about 25% of the variance was genetic and environmental factors accounted for about 75% of the variance (Henn et al., 2004). Further studies showed that stress was the most important environmental factor in the development of depression (Paykel, 2003; Charney and Manji, 2004).

The connection between stress and depression was initially drawn from clinical observations of abnormalities of stress reactivity in depressed patients, including dysfunction of hypothalamic–pituitary–adrenal (HPA) axis (Carroll and Curtis, 1976; Carroll et al., 1976b,c), disrupted cortisol rhythmicity (Sachar et al., 1973; Linkowski et al., 1985) and elevated cortisol levels in plasma (Carroll et al., 1976a), cerebrospinal fluid (Träskman et al., 1980), urine (Scott and Dinan, 1998), saliva (Vedhara et al., 2003), and hair samples (Staufenbiel et al., 2013). These findings solidly underpin a crucial role of cortisol in the development of depression. The link between disruption of the HPA axis and depression also comes from evidence that patients experiencing elevated cortisol levels as a result of Cushing’s disease (Kelly et al., 1983; Sonino et al., 1998) or synthetic glucocorticoid therapy (Brown and Suppes, 1998; Antonijevic and Steiger, 2003; Brown et al., 2004) suffer from depressive episodes. Moreover, some effective antidepressant drugs were found to act on the function of glucocorticoid receptors (GR) to restore the function of HPA axis (Pariante et al., 2001; Okuyama-Tamura et al., 2003; Pariante et al., 2003).

Although dysfunction of the HPA axis has been verified to be closely related to the development of depression, it is still unknown that whether depression promotes dysfunction of the HPA axis or vice versa. In order to elucidate the causal relationship between them, animal models were developed. Among numerous animal models that currently exist, those involving repeated injection are the most promising as this paradigm could provide control over increases in circulating glucocorticoids to manipulate the function of HPA axis (Johnson et al., 2006), which cannot be achieved with other administration methods (e.g., corticosterone pellet implantation or corticosterone in drinking water).

However, previous animal studies involving glucocorticoids are mostly based on rodents, which may pose a major challenge. Rodent brain is phylogenetically distant from human brain (Piper et al., 2011), which makes them differ greatly from humans in anatomy, neurophysiology, function, and behavioral performance and thus limits the degree to which insights derived from the rodents can be applied to understanding human depression (Belmonte et al., 2015). There is another obvious limitation for rodent models that they cannot exhibit the core symptoms of depression (low mood and anhedonia) because of relatively simple brain structure (Song and Leonard, 2005), and they secrete corticosterone in response to stress (Wasser et al., 2000). Compared with cortisol, which is the primary glucocorticoid in both primates and humans, corticosterone has only weak glucocorticoid and mineralocorticoid potencies and is important mainly as an intermediate in the steroidogenic pathway from pregnenolone to aldosterone. By contrast, non-human primates share a common ancestry with humans, and bear strong similarities to humans, such as intricacy of brain organization, details of reproductive biology, complex cognitive capabilities, and great social complexity (Belmonte et al., 2015). As with human beings, non-human primates produce cortisol to cope with stress and can display core depression-like symptoms after exposed to chronic mild stress (Qin et al., 2015a). But what’s even more crucial is that cortisol hyper-secretion can accelerate the occurrence of depressive behaviors in monkeys experiencing more stress (Qin et al., 2016).

Epidemiological studies have indicated that depression occurs nearly twice as frequently in females than in males, which can be ascribed to fluctuations in estrogen associated with reproductive function (Garde, 2007). Therefore, in order to avoid the disturbances of estrogen, male rhesus macaques (Macaca mulatta) were selected in this study and were injected repeatedly with synthetic glucocorticoid to characterize the behavioral and neurobiological consequences of prolonged glucocorticoid treatment, and to further provide insights into the biological mechanisms underlying the link between glucocorticoid and depression.

## Materials and Methods

### Animals

Seven male rhesus macaques, aged 8–10 (8.60 ± 0.60) years old, were randomly selected from Kunming Primate Research Center of the Chinese Academy of Sciences. The animals were singly housed (0.80 × 0.80 × 0.80 m) in a controlled environment (temperature: 22 ± 1°C; humidity: 50 ± 5% RH), with 12 h light/12 h dark cycle (lights on at 07:00 h and lights off at 19:00 h). All monkeys were given commercial monkey biscuits twice a day and were fed with fruits and vegetables once daily. The animals were accommodated in their cages for at least 3 months prior to initial manipulation, and all efforts were made to minimize the monkeys’ suffering. For example, hair samples were taken from the back of the monkeys’ neck using an electric-razor without anesthetic and no animals were sacrificed in this study. Routine veterinary care was provided throughout the experiment by professional keepers and veterinarians.

All animal procedures were approved by the National Animal Research Authority (P.R. China) and the Institutional Animal Care and Use Committee (IACUC) of Kunming Institute of Zoology, Chinese Academy of Sciences.

### Experimental Design

Animals were firstly habituated to experimental procedures to minimize the influences of stress, and then were randomized to glucocorticoid or vehicle group, which was subjected to either prednisolone acetate (3 monkeys) or saline (4 monkeys) treatment. Body weights, HPA axis reactivity and monoaminergic neurotransmitters as well as depression-like behaviors and anhedonia were assessed to analyze the effects of chronic glucocorticoid exposure on monkeys.

### Injection of Prednisolone Acetate and Saline

After 3 months of acclimatization in single cages, the monkeys were injected intramuscularly with prednisolone acetate, which is a synthetic glucocorticoid. The injections (State Medical Permitment Number: H33020824, Drug Specifications: 25 mg/ml) were purchased from Zhejiang Xianju Pharmaceutical Co., Ltd. (China). According to previous studies, the injection dose for rats was 40 mg/kg and this dose reliably induced depression-like behavior in the forced swim test (Kalynchuk et al., 2004; Gregus et al., 2005; Johnson et al., 2006). Using the BSA (body surface area) method, the injection dose for monkeys was calculated (15.89 mg/kg). Saline was used as solvent of prednisolone acetate injection in this study, because it is less painful than sterile water for injection. As a result, the vehicle group was injected with equivalent does of saline. Throughout the experiment, all treatments were performed at 10:00 a.m.

### Blood and Hair Samples Collection

In order to assess the acute HPA axis reactivity, each monkey was sampled 2 ml of blood. Each cage that monkeys lived was equipped with one locking push-pull device and the restraint could be achieved by reducing volumes of the cage. After 15 min of restraint, the blood was sampled from the femoral vein by pulling out the monkey’s leg, with the restraint being the acute stressor. The blood samples were then put into a heparin lithium-treated vacuum collection tube. However, the stress of restraint, blood collection, and the limitation on the volume of blood that could be safely drawn from each monkey, precluded the possibility of repeated sampling of the same monkey. As a result, three blood samples were collected for each monkey every 14 days, with the first, second and third sample obtained on 14, 28, and 42 days after treatment, respectively. As there was an obvious rhythmic change in blood cortisol, all blood samples were obtained at the same time of the day (between 10:30 a.m. and 11:30 a.m.).

Hair samples from all monkeys were collected at the same time of the day (between 10:30 a.m. and 11:30 a.m.) to assess the chronic HPA axis reactivity. Before treatment, each monkey was captured by an experienced technician and taken out of the cage for hair sampling. The monkey was manually restrained, and the hair on the back of each animal’s neck was shaved with an electric razor without the use of anesthetic, with particular attention paid by technicians not to break or damage the skin. After completion of treatment, newly grown hair was shaved as previously described and the hair samples were placed into a small pouch of aluminum foil for protection (Wennig, 2000; Davenport et al., 2006).

### Measurement of Cortisol From Blood and Hair Samples

The blood samples were centrifuged at 8,000 ×*g* for 10 min to isolate plasma, and the hair samples were ground to powder to break up the hair’s protein matrix and to increase the surface area for the extraction of cortisol (Davenport et al., 2006; Feng et al., 2011; Qin et al., 2013, 2015a,b). The cortisol concentration in each blood and hair sample was quantified with a commercial cortisol radioimmunoassay (RIA) kit. In order to minimize the cross-reaction for prednisolone, the cortisol Kit “TFB” (Immunotech, Tokyo, Japan) was used because of its lowest cross-reaction (2.5%) for prednisolone (Horie et al., 2007). The cortisol RIA was performed in triplicate under a double-blind design at the Department of Nuclear Medicine of the Second Affiliated Hospital of the Kunming Medical College. The limit of detection for the cortisol assay was 0.5 μg/dL, and the intra-assay coefficient of variability (CV) for this assay was 2.08%. All the samples were assayed at the same time using the same kit.

### Cerebrospinal Fluid Sampling and Measurement of Monoaminergic Neurotransmitters

Before and after the treatment, all monkeys were sampled cerebrospinal fluid (CSF) under ketamine anesthesia (15 mg/kg) within 10–20 min after their anesthesia. Using a spinal needle, the CSF was obtained through a lumbar puncture. During the procedure, the monkey was positioned in lateral recumbency and a needle was inserted usually between the 3rd and 4th lumbar vertebrae. The CSF fluid was collected into a polypropylene tube and immediately frozen in liquid nitrogen. For purposes of comparison, CSF samples were collected from the same sites. All the collected CSF samples were stored at -80°C until assayed.

Before analysis, CSF sample was centrifuged at 4°C in a high speed freezing centrifuge at 8,000 ×*g* for 10 min. Homovanillic acid (HVA), 5-hydroxyindole acetic acid (5-HIAA) and dopamine (DA) concentrations in the supernatant was quantified by high performance liquid chromatography (HPLC) with electrochemical detection (Yang and Beal, 2011). The CSF samples from a given subject obtained before and after treatment were paired and run in a single assay.

The HPLC system was composed of an Antec LC-110 solvent delivery module, and an Antec Autoinjector AS-110. The separation of HVA and 5-HIAA was performed using an Antec ALF-115 column (C18, 3 μm, 150 × 1 mm). The mobile phase comprising 50 mM monobasic sodium phosphoric acid, 8 mM Sodium chloride, 0.1 mM EDTA, 10.0% (v/v) methanol, 500 mg/L OSA with the final pH adjusted to 6.00 with phosphoric acid, was maintained at a flow rate of 40 μl/min. The separation of DA was performed using an Antec ALF-105 column (C18, 3 μm, 50 × 1 mm). The mobile phase comprising 50 mM phosphoric acid, 50 mM Citric acid, 8 mM Sodium chloride, 0.1 mM EDTA, 12.5% (v/v) methanol, 500 mg/L OSA with the final pH adjusted to 3.25 with phosphoric acid, was maintained at a flow rate of 40 μl/min. The optimal electrical potential settings were: E1 -700 mV and E2 +700 mV. For electrochemical detection, an Antec Decade-11 detector was used. Quantification of the detector signals was achieved by means of peak area integration. Data represent the average of at least two analysis.

### Behavioral Sampling and Analysis

Animal behaviors were video-recorded using a focal follow technique (Altmann, 1974). Before the experiment, fourteen 1 h recordings were collected for each monkey, 1 h each day. Specifically, they were, respectively, collected from 7:00 a.m. to 8:00 a.m., 8:00 a.m. to 9:00 a.m., 9:00 a.m. to 10:00 a.m., 10:00 a.m. to 11:00 a.m., 11:00 a.m. to 12:00 p.m., 12:00 p.m. to 13:00 p.m., 13:00 p.m. to 14:00 p.m., 14:00 p.m. to 15:00 p.m., 15:00 p.m. to 16:00 p.m., 16:00 p.m. to 17:00 p.m., 17:00 p.m. to 18:00 p.m., 18:00 p.m. to 19:00 p.m., 9:00 a.m. to 10:00 a.m., and 14:00 p.m. to 15:00 p.m. After the start of the experiment, the monkeys’ depression-like behaviors were assessed weekly. Once the monkey was observed to become obviously depressed, the treatment was finished and another fourteen 1 h recordings were collected for each monkey at the same time as before.

Each video-recording was scored simultaneously by three observers unaware of experimental design. During the scoring, the observers calculated the duration of specific behaviors by manually starting and stopping the video, and they all agreed on the definition of observed behaviors, including depression-like huddling behavior, environmental exploration, locomotion, stereotypic behaviors and self-grooming. All these behaviors were quantified as frequencies and seconds per hour. The inter-rater correlation coefficient was found to be > 0.90 through SPSS statistical analysis and there were no significant changes (Version 19.0 for the PC) after a period of training.

Depression-like huddling behavior was defined as a fetal-like, self-enclosed posture with the head at or below the shoulders during the waking state (Harlow and Suomi, 1971; Shively et al., 2005). Environmental exploration included tactile exploration of the cage or environments and oral exploration of the cage or environments (Davenport et al., 2008). The locomotion was divided into spontaneous locomotion (defined as any voluntary movement within the cage, including walking, running, jumping and climbing) and reactive locomotion (defined as the locomotion caused by external stimuli) (Rogers et al., 2008). The stereotypic behaviors were defined as frequent, repetitive, and constant postures or behaviors that appear to serve no purpose, including pacing, saluting, somersaulting, weaving and head tossing (Hugo et al., 2003). Self-grooming included any picking, scraping, spreading, mouth picking, or licking of the hair on monkey’s own body (Parks and Novak, 1993).

### Sucrose Preference Test

Anhedonia is a core symptom of depression (Gaillard et al., 2013), and can be evaluated in rhesus monkeys using the sucrose preference test (Paul et al., 2000). During the period of acclimatization, all animals were adapted to a 23 h/day water restriction schedule, and had access to water in two identical bottles for 1 h per day. During the test period, animals were supplied with 1.5% sucrose solution (SIGMA, Aldrich, China, dissolved in tap water) in one bottle, and tap water in the other one. Bottle positions were alternated daily to control for position preference, and the bottles were refilled 30 min after the start of the access period to accommodate the increased consumption. The sucrose concentration was selected based on previous experiments with rhesus monkeys (Paul et al., 2000). The test schedule included 14 days of continuous exposure to sucrose solution and tap water, corresponding to the behavioral sampling period. The test occurred between 9:00 a.m. and 10:00 a.m. The sucrose preference was calculated as ml consumed per kg body weight because the monkey’s water consumption was correlated with its body weight (Paul et al., 2000).

### Statistical Analysis

Data analysis was conducted with the SPSS software package (SPSS Inc., Chicago, IL, United States). The normality of the data was determined using the Kolmogorov-Smirnov test, the results of which demonstrate that they are normally distributed (all *P*-values > 0.05). And the data were further analyzed in separate 2 (groups: predonisolone versus vehicle) × 3 (time: 14th day, 28th day, and 42nd day) or 2 (time: pre-treatment and post-treatment) repeated-measures ANOVAs, with time being the repeated-measure. Further *post hoc* pairwise comparisons (Bonferroni correction) were also made. The alpha level was set at *P* = 0.05, and all *P*-values were generated using two-sided tests. All the data were presented as the mean ± SEM (standard error of the mean).

## Results

### Body Weights

**FIGURE 1:**
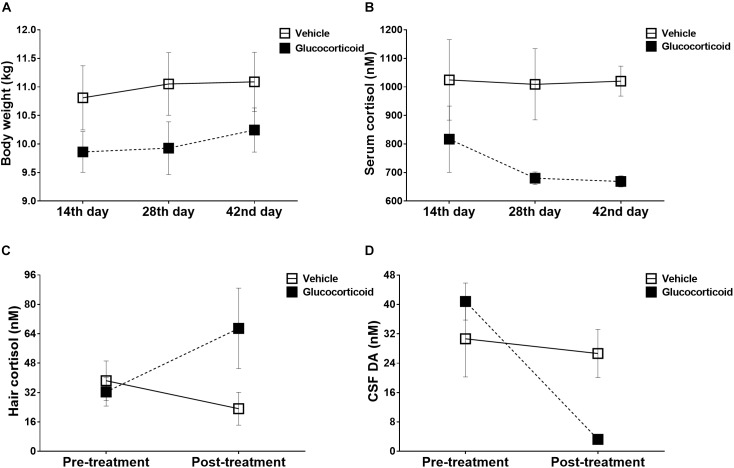
Physiological changes induced by chronic glucocorticoid exposure. Mean (±SEM) physiological changes, including body weights **(A)** and plasma cortisol **(B)**, are depicted for the glucocorticoid and vehicle group on 14th day, 28th day, and 42nd day post-treatment. Chronic glucocorticoid exposure induced no significant changes in monkeys’ body weights throughout the experiment, but the glucocorticoid group exhibited attenuated cortisol reactivity to the acute stressor than the vehicle group. Before and after treatment, the hair cortisol **(C)** and CSF DA **(D)** were also measured to assess the chronic stress reactivity and neurotransmitter function. The glucocorticoid group displayed exaggerated cortisol reactivity and neurotransmitter deficiency compared with the vehicle group.

As shown in Figure 1A, no significant changes over time were observed in monkeys’ body weights during the experiment (*F* = 3.434, *P* = 0.073, η*_p_*^2^ = 0.407) and there were no significant differences between the two groups (*F* = 1.872, *P* = 0.230, η*_p_*^2^ = 0.272), including on the 14th day (*F* = 1.701, *P* = 0.249), the 28th day (*F* = 2.219, *P* = 0.197) and the 42nd day (*F* = 1.482, *P* = 0.278) after treatment. The group by time interaction was also non-significant (*F* = 0.626, *P* = 0.554, η*_p_*^2^ = 0.111). Further *post hoc* pairwise comparisons (Bonferroni correction) showed no significant differences (all *P*-values > 0.05).

### Acute Stress Reactivity

The glucocorticoid group exhibited attenuated cortisol reactivity to the acute stressor compared with the vehicle group (Figure 1B, *F* = 8.419, *P* = 0.034, η*_p_*^2^ = 0.627), and on the 42nd day after treatment, the cortisol level was decreased significantly (Figure 1B, *F* = 30.034, *P* = 0.003). Further *post hoc* pairwise comparisons (Bonferroni correction) showed no significant differences (all *P*-values > 0.05).

### Chronic Stress Reactivity

Although there was no significant difference between the two groups in cortisol reactivity to the chronic stress (Figure 1C, *F* = 1.353, *P* = 0.297, η*_p_*^2^ = 0.213), the glucocorticoid group displayed exaggerated cortisol reactivity as group by time interaction was significant (*F* = 9.678, *P* = 0.027, η*_p_*^2^ = 0.659).

### Monoaminergic Transmitters

During the treatment, the monkeys displayed significant changes over time in the level of dopamine (Figure 1D, *F* = 24.092, *P* = 0.004, η*_p_*^2^ = 0.828), and the group by time interaction was also significant (Figure 1D, *F* = 15.693, *P* = 0.011, η*_p_*^2^ = 0.758). The level of dopamine was higher in the vehicle group than that of the glucocorticoid group on the 42nd day after treatment (Figure 1D, *F* = 8.862, *P* = 0.031). While, no significant differences were found in metabolites of the other two monoaminergic neurotransmitters, including HVA (*F* = 1.334, *P* = 0.300, η*_p_*^2^ = 0.211; vehicle group, pre-treatment versus post-treatment: 160.085 ± 23.278 versus 178.031 ± 41.083; glucocorticoid group, pre-treatment versus post-treatment: 121.236 ± 59.749 versus 95.602 ± 24.181) and 5-HIAA (*F* = 0.138, *P* = 0.726, η*_p_*^2^ = 0.027; vehicle group, pre-treatment versus post-treatment: 61.026 ± 4.987 versus 64.064 ± 5.417; glucocorticoid group, pre-treatment versus post-treatment: 76.099 ± 16.593 versus 87.030 ± 16.774).

### Depression-Like Behavior

**FIGURE 2:**
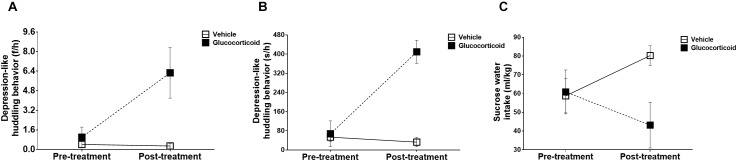
Depression-like behaviors. The monkeys’ depression-like behaviors, including the huddling behavior (**A:** frequencies per hour and **B:** seconds per hour) measuring depressed mood, and sucrose solution intake **(C)** assessing anhedonia, were recorded for 14 consecutive days and the changes before and after treatment were compared between the two groups. Chronic glucocorticoid exposure induced significant changes in external behavioral phenotype, such as increases in the huddling behavior and decreases in sucrose solution intake, which, respectively, represented two core symptoms of depression.

During the experiment, the monkeys’ huddling behaviors exhibited significant changes over time, including frequencies (Figure 2A, *F* = 6.368, *P* = 0.053, η*_p_*^2^ = 0.560) and durations (Figure 2B, *F* = 53.476, *P* = 0.001, η*_p_*^2^ = 0.914), and the group by time interactions were also significant in frequencies (Figure 2A, *F* = 7.003, *P* = 0.046, η*_p_*^2^ = 0.583) and durations (Figure 2B, *F* = 68.111, *P* = 0.0004, η*_p_*^2^ = 0.932). The differences between the two groups were also found to be significant (frequencies: *F* = 14.691, *P* = 0.012, η*_p_*^2^ = 0.746; and durations: *F* = 20.680, *P* = 0.006, η*_p_*^2^ = 0.805). While the frequencies and durations of other behaviors, including environmental exploration, spontaneous locomotion, passive locomotion, stereotyped behaviors and self-grooming, did not display significant changes (all *P*-values > 0.05) except for significant changes over time in durations of reactive locomotion (*F* = 12.400, *P* = 0.017, η*_p_*^2^ = 0.713).

### Anhedonia

There were no significant changes over time in the preference of sucrose water (Figure 2C, *F* = 0.102, *P* = 0.763, η*_p_*^2^ = 0.020), but the group by time interaction was significant (*F* = 10.610, *P* = 0.023, η*_p_*^2^ = 0.680) with the glucocorticoid group consuming less sucrose solution than the vehicle group (*F* = 9.640, *P* = 0.027). However, no significant changes were found in the consumption of tap water (all *P*-values > 0.05).

### Relations of HPA Reactivity and Neurotransmitters to Depression-Like Behaviors

There were no significant correlations between HPA axis reactivity and the levels of dopamine, including the acute stress reactivity (Pearson Correlation Coefficient = 0.470, *P* = 0.288), and chronic stress reactivity (Pearson Correlation Coefficient = 0.704, *P* = 0.077). Further analysis revealed that the acute stress reactivity was not related to the chronic stress reactivity (Pearson Correlation Coefficient = 0.328, *P* = 0.472).

**FIGURE 3:**
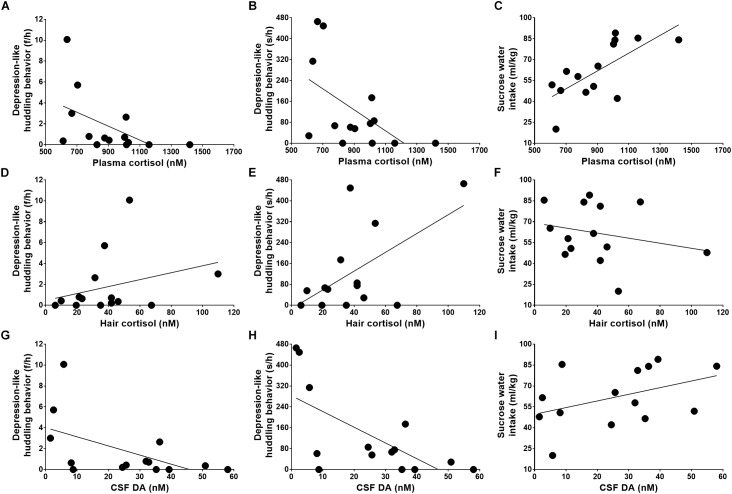
Relations of stress reactivity and dopamine to depression-like behaviors. The depression-like behaviors, including huddling behavior and anhedonia, were caused by attenuated acute stress reactivity **(A–C)**, exaggerated chronic stress reactivity **(D–F)**, and neurotransmitter deficiency **(G–I)**.

Although plasma cortisol was not related to the frequencies of huddling behavior (Figure 3A, Pearson Correlation Coefficient = -0.516, *P* = 0.059), it was strongly related to the durations of huddling behavior (Figure 3B, Pearson Correlation Coefficient = -0.553, *P* = 0.040). Moreover, the plasma cortisol level showed a strong negative correlation with the severity of anhedonia symptoms (Figure 3C, Pearson Correlation Coefficient = 0.699, *P* = 0.005).

Further analysis revealed a moderate positive correlation between the huddling behavior and the cortisol reactivity to the chronic stressor as quantified by hair cortisol (Figure 3D, Pearson Correlation Coefficient = 0.582, *P* = 0.029). The hair cortisol was not found to be related to huddle frequencies (Figure 3E, Pearson Correlation Coefficient = 0.303, *P* = 0.292) and correlated with sucrose solution intake (Figure 3F, Pearson Correlation Coefficient = -0.233, *P* = 0.422), but these correlations displayed weak tendencies.

As analyzed above, the monkeys’ depression-like phenotype was not only caused by abnormal HPA axis reactivity, including both the acute and chronic stress reactivity, but also was related to neurotransmitter deficiency, such as the lower level of dopamine in CSF (Figure 3G, huddle frequencies: Pearson Correlation Coefficient = -0.547, *P* = 0.043; Figure 3H, huddle durations: Pearson Correlation Coefficient = -0.670, *P* = 0.009; and Figure 3I, sucrose water intake: Pearson Correlation Coefficient = 0.422, *P* = 0.133).

## Discussion

It has long been recognized that glucocorticoid plays a crucial role in mediating the pathogenic effects of stress on depression, but clarifying mechanisms are still dependent upon the development of validated animal models. The majority of studies have been focused on rodents, and it has been consistently reported that chronic corticosterone (CORT) injections can induce depressive behavioral manifestations (Marks et al., 2009; Sterner and Kalynchuk, 2010; Xu et al., 2011). However, it is controversial that whether the results obtained from rodents can serve humans because rats and mice lack the enzyme 17 alpha-hydroxylase in their adrenal cortices, so that they produce CORT (Van Weerden et al., 1992). However, the CORT is of minor importance in humans, except in the very rare case of congenital adrenal hyperplasia due to 17 alpha-hydroxylase deficiency (D’armiento et al., 1983). This makes rodents dubious for experiments on the HPA axis, perhaps also for studying the relationship between glucocorticoid and human depression. In humans and other primates, cortisol is the most abundant and naturally occurring glucocorticoid, suggesting research on primates will provide a critical complement on previous rodents’ studies. That is exactly why the macaques were chosen for this study.

To our knowledge, this is the first study using macaques to clarify the mechanisms underlying the causal relationship between glucocorticoid and depression. It was found that chronic glucocorticoid treatment can inflict severe damage on the monkeys’ responses to stress, including both the acute and chronic HPA reactivity. When an organism undergoes stress, cortisol acts to mobilize energy stores and modulate the immune system, preparing it for fighting or fleeing. Blood samples provide a measurement of the cortisol secretion at the time of stress experiencing, and blood cortisol can therefore be used to test the acute HPA reactivity (Maidana et al., 2013). Hair of monkeys grows at an average speed of 1 cm/month, and the cortisol is constantly deposited in the growing hair shaft. This makes cortisol measurement from newly grown hair capturing systemic cortisol response over the period of hair growth, and therefore can serve as a biological marker for the chronic HPA reactivity (Russell et al., 2012). In this study, prolonged glucocorticoid exposure induced blunted cortisol reactivity in response to acute restraint stress but an exaggerated cortisol response to chronic stress experienced during the treatment. This indicated both the acute and chronic HPA reactivity were disturbed as a result of chronic glucocorticoid treatment.

The hyper-secreted cortisol can cross the blood brain barrier to rob the brain of dopamine, a neurotransmitter that plays a critical role in the subjective pleasure associated with positive rewards (Wise, 2008). This is also evidenced by our study that the monkeys exposed to chronic glucocorticoid treatment have decreased levels of dopamine in the cerebrospinal fluid, which induces them showing less of a preference for sucrose solution, a good manifestation of anhedonia. Postmortem investigations, especially the subjects with severe depression, have also demonstrated reduced concentrations of dopamine both in the cerebrospinal fluid and in brain regions that mediate mood and motivation (Dunlop and Nemeroff, 2007). It has also been found that drugs decreasing the dopamine level or the dopamine receptor antagonist can increase the duration of huddling behavior in monkeys (McKinney et al., 1971; Rosenzweig-Lipson et al., 1994).

Chronic glucocorticoid treatment caused severe damages on stress reactivity and neurotransmitter systems, which induced significant changes in external behavioral phenotype, such as increases in the huddling behavior reflecting depressed mood, and decreases in sucrose solution consumption reflecting anhedonia. These are two core symptoms of human depression. However, this behavioral depression was not induced by decreases in exploration, locomotion, stereotyping and self-grooming, as these behaviors did not change significantly throughout the treatment. This was also not the result of poor health caused by glucocorticoid treatment as no significant changes over time were observed in monkeys’ body weights. Although systemic glucocorticoids play an integral role in the management of many inflammatory and immunologic conditions, there are side effects. Prolonged glucocorticoids treatment commonly causes weight gain and redistribution of adipose tissue. The body weights of monkeys exhibited slight increases, but no significant changes were observed. This is possibly because the glucocorticoid treatment was relatively short in duration (only 7 weeks). The monkeys’ depression had become obvious before a significant weight gain. Further correlation analysis showed that this behavioral depression was caused by abnormal HPA axis reactivity, including both acute and chronic stress reactivity, and it was also related to neurotransmitter deficiency, such as a decreased level of dopamine in CSF.

The behavioral alterations were not significant until 6 weeks after the first daily treatment, and the data justified the conclusion that prolonged glucocorticoid exposure can induce depression-like phenotype in rhesus macaques. The application of this primate model can help clarifying the role of glucocorticoid in stress-induced depressive disorders, as well as screening for novel therapeutic targets and specific preventive strategies for hypercortisolemia-induced depression.

## Author Contributions

All authors listed have made a substantial, direct and intellectual contribution to the work, and approved it for publication.

## Conflict of Interest Statement

The authors declare that the research was conducted in the absence of any commercial or financial relationships that could be construed as a potential conflict of interest.
